# The gut microbiome meets nanomaterials: exposure and interplay with graphene nanoparticles

**DOI:** 10.1039/d3na00696d

**Published:** 2023-10-18

**Authors:** Olga Wojciechowska, Adele Costabile, Małgorzata Kujawska

**Affiliations:** a Department of Toxicology, Poznan University of Medical Sciences Rokietnicka 3 Poznan 60-806 Poland kujawska@ump.edu.pl; b School of Life and Health Sciences, University of Roehampton London SW15 4JD UK

## Abstract

Graphene-based nanoparticles are widely applied in many technology and science sectors, raising concerns about potential health risks. Emerging evidence suggests that graphene-based nanomaterials may interact with microorganisms, both pathogens and commensal bacteria, that dwell in the gut. This review aims to demonstrate the current state of knowledge on the interplay between graphene nanomaterials and the gut microbiome. In this study, we briefly overview nanomaterials, their usage and the characteristics of graphene-based nanoparticles. We present and discuss experimental data from *in vitro* studies, screening tests on small animals and rodent experiments related to exposure and the effects of graphene nanoparticles on gut microbiota. With this in mind, we highlight the reported crosstalk between graphene nanostructures, the gut microbial community and the host immune system in order to shed light on the perspective to bear on the biological interactions. The studies show that graphene-based material exposure is dosage and time-dependent, and different derivatives present various effects on host bacteria cells. Moreover, the route of graphene exposure might influence a shift in the gut microbiota composition, including the alteration of functions and diversity and abundance of specific phyla or genera. However, the mechanism of graphene-based nanomaterials' influence on gut microbiota is poorly understood. Accordingly, this review emphasises the importance of studies needed to establish the most desirable synthesis methods, types of derivatives, properties, and safety aspects mainly related to the routes of exposure and dosages of graphene-based nanomaterials.

## Abstract 2

We provide a brief overview of graphene-based nanoparticles, their usage and characteristics. We discuss experimental data from *in vitro* screening tests on small animal experiments related to exposure and the effects of graphene nanoparticles on gut microbiota.

## Graphical abstract

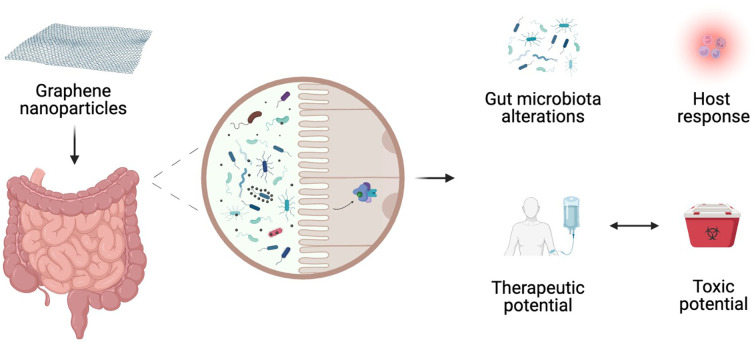

## Introduction

**Fig. 1:**
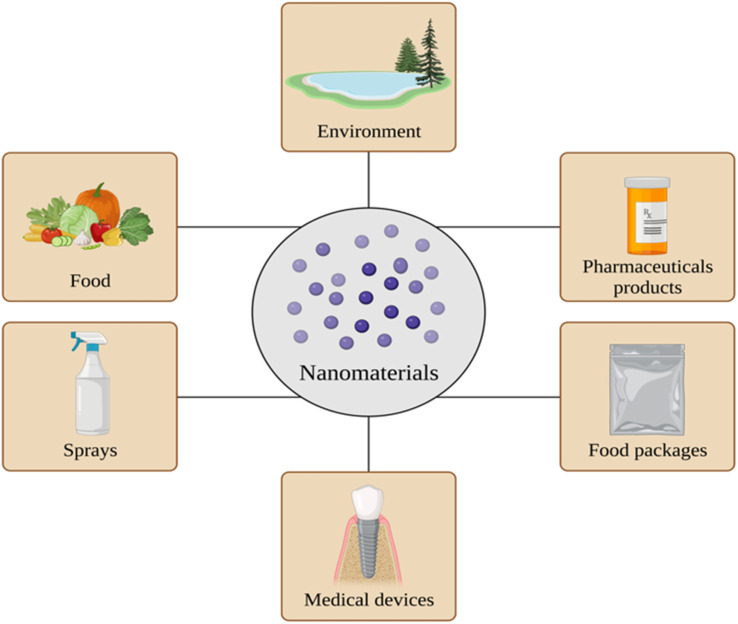
Overview of the sources and usage of nanomaterials. Created with https://www.BioRender.com.

There has been significant growth in nanotechnology and nanoscience in the twenty-first century. It found its way into many sectors of technology and science. Nanoscience is the study of particles ranging between 1 and 100 nm, while nanotechnology represents their application in practical use.^1^ The global market of nanomaterials was considered to be approximately 8.5 billion dollars, with an expected annual growth rate of 13.1% between 2020 and 2027.^2^ This rapid growth of the values of nanomaterials and the application of nanotechnology is based on dynamic and effective changes in the industry and adaptable usage of nanomaterials in the medical sector, pharmaceutics, agriculture, food industry, construction, cosmetics, the energy sector, electronics, *etc.*^3,4^ (Fig. 1). The emerging market of nanomaterials applied in the health sector is especially significant. Nanomaterials might be considered as a form of treatment and/or diagnostics. Their characteristics may impact the drug formulation, delivery, or route of drug administration.^5^ However, taking into account the widespread distribution of nanomaterials applied and their exposure to the human body, there is deliberation about how they influence the organism and what potential health risks they might be causing.

Because of their unique properties, carbon-based nanoparticles are widely applied in the biomedical sector, including carbon nanotubes, graphene and its derivatives, nanodiamonds, fullerenes, and others.^6^ Among them, graphene-family materials deserve distinctive interest. Graphene is composed of a single layer of carbon atoms arranged in a two-dimensional, hexagonal honeycomb lattice.^7^ The increasing interest in graphene-based nanoparticles is based on their distinctive physicochemical properties. Graphene and its derivatives are characterised by electrical conductivity, biocompatibility as well as mechanical strength.^7–9^ These properties enable their application in medical diagnosis, cancer therapy, as well as drug, molecule, or gene delivery.^10,11^ Moreover, one of many opportunities also includes the treatment of neurodegenerative diseases like Alzheimer's or Parkinson's.^12^ Nano-stem cell therapy holds promise to cure Parkinson's disease,^13^ and graphene-based treatment directly affecting proteinopathies is also projected.^14^ Despite these advantages, there are also concerns about potential health risks and the influence graphene may have on the human body. Taking into consideration different routes of exposure to graphene-based nanoparticles, like oral, transdermal, and nasal inhalation, various tissues or cells might be affected.^15^ Several *in vivo* and *in vitro* studies examined the impact of the exposure to graphene-based nanomaterials and their potential health risk (reviewed in ref. 16–19). Due to the oral exposure of the graphene-based nanoparticles, which also results from inhaled nanomaterials being absorbed after mucous membrane clearance, not only is the host gastrointestinal tract (GI) exposed to potential nanotoxicity, but the alteration in the microbial composition in the gut is also considered.^20,21^ Therefore, thorough and objective analysis is required to evaluate the nanoparticles' health impact and potential toxicity.

Human intestines are inhabited by numerous microorganisms, such as bacteria, archaebacteria, fungi, and viruses. This composition is defined as gut microbiota.^22^ There are trillions of bacterial cells, which are the main components of the ecosystem. A distinctive interaction between the host organism and microbiota might be observed. The gut microbial composition plays a role, *i.e.*, in digestion, protein, lipid, carbohydrate, energy metabolism, immune and inflammation response, and epithelial function.^22,23^ As a result of their unique characteristics and functions, an appropriate microbial composition is crucial to human health and well-being. The individual composition of bacteria is influenced by numerous factors, including delivery, diet, environment, living conditions, physical activity, diseases or disorders, and drugs.^24–26^ Moreover, the exposure to the nanomaterials, including graphene-based nanomaterials, might also alter the gut microbiota composition. The exposure might cause disturbances in the growth and functioning of the bacteria from specific species or genera, leading to dysbiosis exhibited by reshaped symbiotic relations between the host and microorganisms.^27–29^ Dysbiosis could manifest GI symptoms and a systemic impairment of homeostasis.^30^ Hence, evaluating the impact and nanotoxicity of the exposure of graphene and its derivatives on the bacterial organism is crucial. On the other hand, understanding fundamental behaviours and the fate of graphene nanomaterials in biological hosts, including degradation, transformation and bioavailability, is also critical for accurately assessing their toxicity in organisms.^31^

Taking into consideration the issues mentioned above, the aim of this review is to explore the interplay between graphene nanomaterials and the gut microbiome. The review emphasises the analysis of the studies focused on the exposure of the nanomaterials and mechanisms underlying their influence on bacterial composition. Different types of graphene-based nanomaterials have been described. Also, an extensive description of the gut microbiome has been provided. Finally, the review discusses the available *in vitro* and *in vivo* studies on graphene nanomaterials and microbiota.

## Nanomaterials

Nanomaterials are a wide group of particles applied in various sectors. Due to the development of nanoscience, new types of nanomaterials have been engineered. However, in addition to these, nanoparticles are also naturally present in the environment. Because of the outspread of nanomaterials, hazard assessment must be acknowledged.^19,32,33^

### Presence and usage of nanomaterials

As mentioned before, nanomaterials are integrated into numerous branches of industry, depending on their properties, design, size, or reliability. Considering toxicology assessment, specifically, food and packages, sprays, medical devices, and pharmaceutical products are pointed out as a source of nanoparticle exposure^34–36^ (Fig. 1).

Food is considered to be a major source of exposure to nanoparticles *via* oral administration. Their potential sources might be engineered as well as natural nanomaterials, additives, packages, formatted during food processing, and ingested by animals from the environment.^37^ Specifically food contact materials have been targeted as a possible source of nanomaterials in food, including inappropriate storage, usage and the production process of food packages and containers.^38^ A prominent pathway for the elimination of nanoparticles ingested is faecal excretion. However, due to their different physio-chemical properties, they could interact with host cells as well as gut microbiota.^39,40^ Most nanostructures are trapped in innate immune cells correlated with toxicity, particularly inflammation, after being ‘coronated’ with host proteins and other biomolecules on their surfaces.^41,42^ Moreover, nanomaterials have been observed to accumulate in the liver, kidneys, and other organs.^43^ Nevertheless, the distribution, accumulation, possible aggregation, bioavailability, and cytotoxicity of nanomaterials from food are not yet fully discovered. Also, there is a necessity for validated and standardised methods and tools for checking the risk assessment of nanomaterials in food.^44^

Sprays, including cosmetics or cleaning products, pose potential inhalation exposure. Some studies link nanomaterial exposure from sprays to acute or chronic lung toxicity, airway inflammation, or lung cell cytotoxicity.^45,46^ Health threats are affected mainly by the particles' size, shape, and dose. The main limitation of the risk assessment is the dose metrics of the exposed nanoparticles due to the misuse of products.^44^

In the medical sector, nanoparticles are widely used as a part of medical devices and pharmaceuticals. The nanoparticles are being used as visual prosthesis, cardiovascular stents, dental implants, osteosynthesis materials, and orthopaedic implants.^47,48^ The primary health concern is regarded as material degradation and the inflammation of the peri-implant environment, including membranes and tissues.^49^ The employment of toxicokinetic studies is needed, aiming for the detection and quantification of long-term use and release in the human organism.^44^ Due to their properties, nanomaterials are used as carriers for drugs, molecules, and other functional substances. The particles are employed to improve the formulation, encapsulation, dispersion, and suspension of insoluble compounds and taste, and release of the drug.^50^ They also pose barriers from environmental, abrasive factors of the GI tract.^51^ However, apart from food, orally or nasally administered drugs and supplements are a possible health concern for the GI tract and gut microbiota. Therefore, similar concerns are present in the research regarding the ingestion of nanoparticles from pharmaceuticals like from food.^52^ The main goals of future research on nanoparticles in pharmaceuticals include the reduction of toxicity while maintaining their beneficial properties and greater biocompatibility, and more specific targeting.^53,54^

The presence of nanoparticles in the environment is natural and also a consequence of anthropological activities. Nanoscience and the development of nanomaterials are spreading rapidly in numerous sectors. Apart from the intentional utilisation of nanomaterials, humans and other organisms might be exposed to them in the environment.^55^ Increased usage of nanomaterials introduces a great number of particles into the ecosystem, including air, water and soil. The exposure could be related to using nanomaterials in the environment and waste from other sectors. The amount of released particles into the ecosystem could not be accurately estimated. Nanomaterials are emerging as contaminants of environmental concern. Therefore, there is a need for the development and evaluation of methods and tools regarding the detection and measurement of nanomaterials in the environment. Moreover, further studies should investigate the assessment of the toxicity of the nanoparticles. The gap in research relates to difficulties in the evaluation of concentration and impact on complex environmental samples.^56,57^ Simultaneously, the development of nanotechnology might have beneficial implications for the environment. Therefore, the risk assessment should also include possible positive effects on the environment.

### Graphene and its derivatives

Graphene is an allotrope of carbon consisting of a single atom thick sheet, sp^2^ bonded and arranged in a two-dimensional honeycomb lattice structure. Layers of graphene are piled together to form graphite, making it a three-dimensional structure.^7–9^ Graphene is considered a hydrophobic, electrically conductive semimetal. It is described as a light, but thermally and mechanically strong material. Due to its properties, it has been applied in water filtration, energy storage, biosensors, and solar cells.^58^ Moreover, the unique properties of graphene nanomaterials make them suitable for drug delivery systems as carriers of small-molecule drugs, genes, or antibodies. These mechanisms are possible because of the capability of graphene-based nanomaterials to cross the blood–brain barrier.^10,11,59^ Graphene-based materials vary in layer number, lateral dimension, surface chemistry, and defects or quality of the individual graphene sheets. A few graphene derivatives might be distinguished, including graphene oxide (GO), reduced graphene oxide (rGO), and graphene quantum dots (GQDs).^58^ GO is composed of carbon, oxygen (with a C/O ratio between 1.5 and 2.5), and hydrogen atoms. GO is chemically modified graphene derived from the oxidation and exfoliation of graphite, which leads to increased interlayer spacing. The oxygen groups located on the surface provide reaction sites for linking proteins, enzymes, peptides, nucleic acids, molecules and cells.^60–62^ rGO is produced from GO by thermal, chemical, or electrochemical reduction to decrease oxygen content. Despite having some properties similar to graphene, it usually contains more defects and has a lower quality. Regardless, rGO could still be administered in various applications suitable for graphene uses.^61,63,64^ GQDs are described as graphene-based nanoparticles with unique properties like biocompatibility, photostability, and membrane permeability.^65^ As quantum dots, they can be characterised as semiconductor particles with different optical and electronic properties than those of larger particles because of quantum mechanics.^66^ Graphene quantum dots usually have a size range below 20 nm diameter and might be fabricated by fragmentation of graphene sheets.^65,67^

## Gut microbiota

### Normal microbial composition

The term “gut microbiota” is regarded as the collection of intestinal bacteria, archaebacteria, fungi, and viruses. The density of bacteria in the colon is estimated to be 10^11^–10^12^ cells per ml.^68^ The gut microbiome encodes more than 3 million genes, while the human genome is estimated to include about 23 000 genes. The studies suggest that over 1000 different bacterial species can be isolated from the intestines of a healthy person; however, the number might be even higher.^22,68,69^ The diverse gut microbiota living in symbiosis with a host can be considered as a “metaorganism”.^70^ Gut bacteria are a notable part of digestion – they play an important role in the extraction, synthesis, and absorption of many nutrients and metabolites, including lipids, amino acids, bile acids, and short-chain fatty acids (SCFAs). Intestinal microbiota participates in immune functions, inhibiting the growth of pathogens and preventing their invasion by maintaining the integrity of the intestinal barrier.^22,71–73^ Intestinal microbes also influence neurological signalling, modify drug metabolism and action, eliminate toxins and produce numerous compounds that affect the host homeostasis and health.^74^

Most bacteria in the intestines are anaerobic.^75^ The dominant phyla of intestinal microbes are Firmicutes and Bacteroidetes, followed by Actinobacteria, Proteobacteria, Verrucomicrobia, and Fusobacteria. In general, the microbiota of a healthy adult consists mainly of Firmicutes and Bacteroidetes species, which make up about 90% of the composition. Firmicutes phyla consist of more than 200 different genera, such as Clostridium (usually about 95% of the phyla), Lactobacillus, Bacillus, Enterococcus, and Ruminococcus. The Bacteroidetes phylum is represented mainly by Prevotella and Bacteroides. Other less abundant phyla are Actinobacteria, represented mostly by Bifidobacterium and Verrcomicrobia, usually covered by the genus Akkermansia and Proteobacteria – by Escherichia.^22,68^

Although the overall individual profile remains constant, the gut microbiota shows both temporal and spatial alterations in the distribution at the genus level and beyond. The diversity of the microbial community and its abundance of species enables a balanced and healthy composition of intestinal microbes.^30^ While the composition remains relatively resistant to acute disturbances, constant exposure to various stress factors might lead to dysbiosis and pathogenic bacteria overgrowth. Stress factors are usually related to the modern lifestyle, including Western diet, food additives, heavy metals, pesticides, mycotoxins, pollutants, and usage of antibiotics and other drugs.^76,77^ The alterations of microbial composition might depend on the type of drugs, dose, route of administration, duration of the exposure, and pharmacological action.^26^

### The key roles of gut microbiota

Numerous roles of bacterial composition in host health and disease are undeniable. Bacteria are engaged in many metabolic and immune pathways of the host organism. The richness and diversity of the microbial community are important factors in human health.^69–72^

Gut microbiota plays a role in the process of digestion, enabling the extraction and absorption of nutrients, including lipids, amino acids and carbohydrates. Numerous bacterial genera have the ability to produce vitamins, including B-group and K.^69^ Bacteria synthesise SCFAs during the fermentation of non-digestible carbohydrates. The acids are involved in colonic homeostasis, appetite regulation, intestinal barrier integrity maintenance, and metabolism regulation.^78^ Moreover, gut microbiota contributes to the development, training and functions of the immune system. Microbial composition prevents pathogenic invasion and infections. Commensal bacteria support the intestinal epithelium integrity, decrease colonic pH, secrete antimicrobial peptides and compete with opportunistic genera for available nutrients.^79^ What is more, the gut microbiota is involved in the bidirectional communication between the gut and the brain. Growing evidence supports microbiota influence on the gut–brain axis through neuroendocrine, metabolic, immunological and neuroanatomical pathways. Bacteria are involved in the synthesis and release of neurotransmitters, including serotonin, dopamine or γ-aminobutyric acid (GABA) and other bacterial metabolites. Thus, the microbial composition in the GI might result in specific neurochemical and behavioural effects on the host.^74^ Accordingly, an unvaried and low-taxa microbiota may present a decrease in the functions and efficacy. Dysbiosis may contribute to the health of the host and be a risk factor for many diseases.^71^ Alteration in the microbial community has been observed during the incidence of obesity, type 2 diabetes, inflammatory bowel diseases, irritable bowel syndrome, depression, non-alcoholic fatty liver disease, celiac disease, neurodegenerative conditions, including Parkinson's and Alzheimer's disease and more.^25,29,71,80^ However, research is still inconclusive on whether dysbiosis is the risk factor and cause of health deterioration or the consequence. The connection of gut dysbiosis to health outcomes demonstrates the importance of diverse and rich gut microbiota.

### Effect of graphene on bacterial pathways

**Fig. 2:**
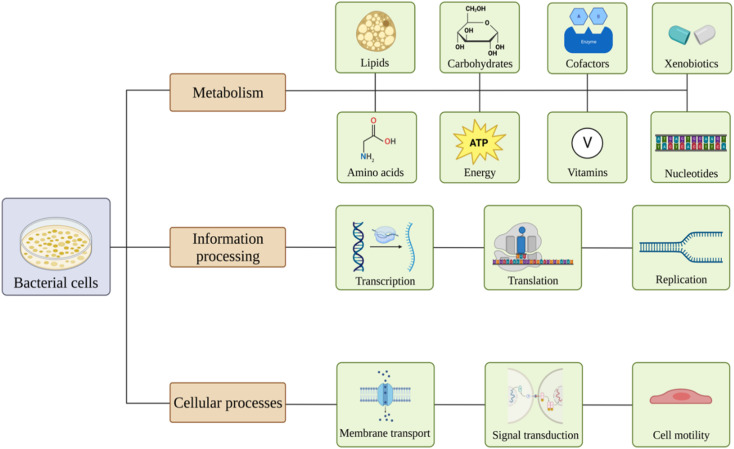
Overview of the effects of nanomaterials on bacterial pathways. Created with https://www.BioRender.com.

Nanomaterials are believed to have various effects on bacterial pathways^81^ (Fig. 2). Exposure might lead to alterations in carbohydrates, lipids, amino acids, nucleotides, vitamins, xenobiotics, cofactors, and energy metabolism. Also, nanoparticles may affect genetic information processing, including transcription, translation, folding, sorting, replication, and repair of the material. Membrane transport, signal transduction, and cell motility could also be influenced.^82–86^

Graphene-based nanomaterials also have effects on bacterial cells. The antibacterial properties are time and concentration-dependent and vary between different graphene derivatives.^87,88^ Even though there are several possible variants of graphene interaction with bacterial cells, the exact cytotoxicity is still unclear.^87^ One of the proposed mechanisms of cell damage by GO exposure is physical damage due to the sharp edges of the particles. Breaking up the integrity of the membrane causes leakage of the cell components into the environment and the disintegration of the bacteria.^89,90^ The destruction of the bacterial cell membrane is also associated with a possible exposure of rGO.^91^ Induction of oxidative stress and genetic material damage might be related to GO or rGO.^92,93^ GO is also a possible cause of the isolation of the bacterial cell from its external environment. The mechanism inhibits bacterial access to nutrients and their proliferation.^94^

Therefore, as graphene-based nanoparticles might be ingested intentionally or unintentionally or swallowed during nasal administration, as they reach the intestines, they might be interacting with the gut community.^95^ The influence of the particles might include alteration in the diversity and abundance of gut bacteria and the intestinal environment.^96^ Thus, a thorough analysis of the *in vitro* and *in vivo* studies of the impact of graphene and its derivatives on the intestinal environment and gut microbiota is critical in determining the particles' toxicity and potential health consequences.

## The influence of graphene-based nanomaterials on the gut microbiota

As stated above, gut microbiota plays an important role in human health and disease. There is a strong correlation between microbial activity and digestion, immunity, metabolism, and the nervous system functions of the host.^97^ Several studies examined the impact of the exposure to graphene-based nanomaterials on the modulation of gut composition and their potential health risks. Therefore, there is a possibility that intentional or unintentional exposure of graphene nanoparticles might influence the host health.^17,18^

### *In vitro* model studies

The interaction of pristine graphene with commensal bacteria, including *Lactobacillus acidophilus*, *Bifidobacterium longum*, and *Escherichia coli*, was evaluated using a bioreactor rotary cell culture system, which prevented sedimentation and therefore enabled a continuous interaction of the tested nanomaterial with the bacterial cultures. The results showed that the growth of lactic acid-producing *L. acidophilus* was promoted by 24 h of continuous exposure to graphene in a dose-dependent manner, while there was no effect on the growth of *E. coli* and *B. longum*.^20^ Due to modulatory activity on the intestinal immune system and gut barrier functions,^98^ the stimulatory effect of graphene on the probiotic *L. acidophilus* can be considered as a beneficial effect. In the faecal samples obtained from healthy male rats, incubation with graphene caused a significant two-fold increase in aerobic and anaerobic bacterial counts (expressed as colony forming unit; CFU) during the first 3 h of exposure. However, after 24 h of continuous exposure, a 120% decrease in aerobic bacteria CFU at the highest concentration (100 μg mL^−1^) of pristine graphene was noted. Moreover, bacterial composition in the faecal samples was affected with significant alterations of 15 taxonomic groups. The expanded quantity of butyrate-producing genera was correlated with an increased concentration of butyric acid after the exposure.^20^ Recently, researchers confirmed that GO-treated gut bacteria produced substantial amounts of butyrate (∼1.1 mM) and a low acetate level, while propionate, valeric and caproic acids were not detected.^31^ Strong evidence exists that butyrates contribute to maintaining intestinal homeostasis through a multifaceted approach, including modulation of genetic expression and signalling pathways. Butyrate is used to generate energy in colonocytes, maintain the intestinal anaerobic environment *via* the transcription factor hypoxia-inducible factor, support the intestinal barrier by regulating the expression of claudin-1 and synaptopodin, limit inflammation cytokines (IL-6 and IL-12), and inhibit tumour pathways (Akt/ERK, Wnt, and TGF-β signalling).^22,99^ Given the ability of butyrates to inhibit the inflammatory response and enhance the gut barrier and GI motility, butyric acid-producing anaerobic bacteria are considered a novel probiotic treatment approach for inflammatory bowel disease (IBD)^100^ and the potential management of Parkinson's disease.^101^ By necessity, graphene-assisted advances in pharmaceutical applications of these probiotics should include studies on the dose- and time-dependent effect of graphene nanoparticle exposure on butyrate-producing bacteria.^20^ In this context, a recent discovery that gut microorganisms fermented GO into a source of bioavailable butyrate is significant. The GO fermentation process was attributed to the activation of bacterial enzymes involved in the pyruvate pathway, including hexokinase, pyruvate kinase, pyruvate dehydrogenase, butyrate kinase and butyryl-CoA:acetyl-CoA transferase as well as increased amounts of acetyl-CoA and butyryl CoA in the gut bacteria.^31^

The impact of graphene derivative GO on five human gut bacteria – *Bifidobacterium adolescentis*, *Lactobacillus acidophilus*, *Escherichia coli*, *Enterococcus faecalis*, and *Staphylococcus aureus* was also studied.^102^ The obtained results support good biocompatibility and low cytotoxicity of GO to these bacteria. Real-time PCR revealed that GO promoted bacterial proliferation. The exposure to GO resulted in an especially rapid increase in the abundance of *B. adolescentis* compared to other genera. A 13 h incubation of 100 μg mL^−1^ GO resulted in a significant, more than three-fold increase of *B. adolescentis* compared to the control group. The observed increase was 2.5, 2.3, and 1.7-fold in *E. coli*, *S. aureus*, and *L. acidophilus*, respectively. Membrane potential measurements of the tested bacterial cells did not reveal depolarisation, supporting a lack of any adverse effect of GO on its integrity. What is more, GO sheets enhanced the antagonistic activity of *B. adolescentis* against the pathogenic strains of *E. coli* and *S. aureus*. The observed good biocompatibility and low cytotoxicity of GO make the nanomaterial considered for biomedical applications as drug carriers for intestinal systems. The enhancement of *B. adolescentis* proliferation and its antagonistic properties against *E. coli* and *S. aureus* of GO might be a basis for therapeutic approaches.^102^

A more recent study in *in vitro* simulated digestions and colon reactor setup showed that exposure to low (25 mg L^−1^) concentration GO decreased taxonomic diversity and the abundance of the Bacteroidota phylum, causing a shift in the ratio of Firmicutes to Bacteroidota (F/B).^103^ Importantly, *Bacteroides* spp. promotes gut homeostasis by forming host immunity and preventing pathogenic colonisation, and metabolises a wide range of polysaccharides important for the optimal uptake of energy of the host.^104^ Indeed, the increased F/B ratio was reported to be associated with higher energy reabsorption and obesity in mice^105^ and the prevalence of an irritable bowel syndrome subtype.^106^ The drastic increase in the F/B ratio following exposure to 25 mg L^−1^ GO was reported to subsequently affect downstream products of microbial metabolism with impaired production of metabolites such as 5-hydroxyindole-3-acetic acid and an essential neuroactive GABA metabolite.^103^ Surprisingly, at the high (250 mg L^−1^) GO concentration, the increased taxonomic diversity with the phyla Bacteroidota's predominant composition was observed. Moreover, differential abundance analysis showed that three genera of Bacteroidota (Bacteroides, Dysgonomonas, and Parabacteroides) were more abundant after GO exposure at 250 mg L^−1^. Nevertheless, community composition and metabolome profiles at a high GO concentration were concluded to be similar to those of the control.^103^

### *In vivo* model studies

#### Studies on small animal screening models

Whole-animal testing based on small animal models fulfils the requirements for large-scale screens; however, to answer fundamental questions of pharmacology and toxicity, mammalian experiments are still required.^107^ The dietary exposure to monolayer graphene powder (GR), GO, and rGO for twenty-one days has been reported to cause dysbiosis of intestinal microbiota in zebrafish. The diversity of the intestinal microbial community was found to be affected only in the GR group, indicated by the decreased Shannon's H index. However, intestinal microbiota composition was altered in all experimental groups. At the phylum level, the abundance of Bacteroidetes in GR and GO groups was significantly lower than in the control. Moreover, a significant decrease in the abundance of the genus Pseudomonas was observed in all graphene groups. Aeromonas abundance declined, whereas that of Cetobacterium increased following GR and rGO exposure, respectively.^108^ Dysbiosis with reduced bacterial diversity of Bacteroidetes has been shown to be associated with the release of pro-inflammatory mediators in the intestine in IBD.^109,110^ Commensal bacteria from the Bacteroidetes phylum produce sphingolipids, which are structural membrane components and signalling molecules that regulate inflammation and immunity and therefore play a critical role in maintaining homeostasis and symbiosis in the intestine in patients suffering from IBD.^111^ Accordingly, morphological changes in the intestinal tissue and the disintegration of the cell boundaries in GR- and GO-exposed zebrafish with the subsequent decline in Bacteroidetes' abundance have been observed. In contrast, the abundance of probiotic Lactobacillus boosted with around a 16-fold increase noticed in these groups. Interestingly, none of the tested graphene nanomaterials affected the body length and weight of the zebrafish.^108^

The alteration in the gut microbiome profile and community caused by the exposure of GO was also demonstrated in the amphibian *Xenopus laevis* model.^112^ Compared to the control group, a transient decrease in total microbial abundance alongside a shift in the gut composition was observed after two days of exposure to GO. More prolonged exposure to low GO concentrations led to a significant increase in the relative abundance of the Proteobacteria phylum and a decrease in the phylum Fusobacteria, resulting in an increase in the F/B ratio of *X. laevis* tadpoles. The shift was suggested to contribute to the growth inhibition of GO-exposed tadpoles.^112^ This is due to the metabolic capacities of these phyla, and the imbalanced F/B ratio has been demonstrated to be associated with the development of metabolic syndrome or IBD.^113^ At the genus level, the relative abundance of *Bacteroides fragilis* from the Bacteroidetes phylum was reported to increase following GO exposure. Noticeably, GO accumulation in the tadpoles' intestines was observed, which facilitated direct interactions of the graphene nanoparticles with gut bacteria.^112^ On this note, *Bacteroides fragilis* toxin is known the pro-carcinogenic inflammatory cascade in colonic epithelial cells.^114^ This may explain the genotoxicity accompanied by intestinal bacteria modifications following GO exposure in amphibians.^112,115,116^

In contrast, the study performed on *Caenorhabditis elegans* investigated the effect of *Lactobacillus bulgaricus* on GO toxicity.^117^ The primary proposed mechanism of GO-induced cellular toxicity is oxidative stress.^118,119^ Oxidative stress has been demonstrated in the intestines of zebrafish exposed to both GR and GO, which was attributed to decreased antioxidant capability.^108^ The pretreatment with *L. bulgaricus* – lactic acid bacteria prevented the toxicity of 24 h exposure to GO in nematodes by inhibiting intestinal ROS production. This effect was accompanied by improved defecation and locomotion behaviour in nematodes. The administration of the probiotic bacteria affected the pattern of GO distribution, which was restricted only to the primary target organs like the intestine and pharynx. As *L. bulgaricus* pretreatment was associated with maintaining the integrity of the intestinal barrier in GO-exposed nematodes, this mechanism was suggested to contribute to suppressed translocation of GO into the secondary targeted organs.^108^ Significantly, the decreased distribution of GO to germ cells in the reproductive organs could protect these organs^117^ from the genotoxic effect of GO.^120^

#### Rodent model studies

Several studies on graphene-based nanoparticle exposure include work on small mammals, including mice or rats. Orally administrated GO at a dose of 120 mg kg^−1^ on mice has been demonstrated to impair the colon ultrastructure on the 16th day. Even though the cell membranes remained intact, a tendency of apoptosis was observed. The microvilli were arranged unevenly, sparsely, and shrunk locally. The damage to the colon was positively correlated with the dose of GO.^121^ The loss of integrity of the intestinal barrier and related uncontrolled flux of antigens across the barrier challenge the immune system, thus affecting the host-microbial balance.^122^ Accordingly, a shift in the gut microbiota composition was attributed to the decreased abundance of the Firmicutes phylum and the genus of Xanthobacteraceae, Enterobacteriaceae, Enterobacterales, and Alistipes, while Bacteroidota abundance was increased in the GO-exposed mice. Moreover, β diversity was declined.^121^ The GO-induced altered gut microbiome can be accompanied by placenta barrier dysfunction. In pregnant mice, GO administration at a dose over 10 mg kg^−1^ decreased bacterial community richness and community diversities. A decrease in the abundance of Cyanobacteria, Chliroflexi, and Latescibacteria phyla, whereas an increase in Euryarchaeota was observed. Moreover, the shift in the F/B ratio was noted. The study suggests that the oral administration of GO impacted pregnant mice and fetuses. The dysbiosis was connected with placenta barrier dysfunction and related pregnancy complications. Euryarchaeota and Firmicutes abundance was negatively correlated with the expression of tight junction factors on mRNA and protein levels.^123^ Moreover, the dysbiotic gut microbiome is known to induce abnormal immune responses and intestinal barrier destruction, which can facilitate the translocation of pathogenic bacteria to the intrauterine cavity, thus eliciting inflammation and contributing to placenta barrier dysfunction.^124^ Indeed, the increased number of abortions, resorbed embryos, and dead fetuses in the graphene groups was recorded. Also, the lower weight of the mother was positively correlated with the lower weight of the fetus.^123^

Another study investigated the impact of different doses of pristine graphene on the microbial community and the related mechanism of bacterial resistance in the mouse gut.^125^ The 4 week oral exposure to graphene increased the diversity of gut bacteria, with the highest effect at a dose of 1 μg day^−1^. In this group decreased abundance of Gram-positive bacteria Lactobacillus and Mycoplasma, which lack an outer cell membrane, was observed, while Gram-negative Prevotella, Anaeroplasma, and Paraprevotella had a higher abundance. The shift in gut microbial composition might be due to graphene-induced oxidative stress and damage to cell membrane integrity. The outer membrane of Gram-negative bacteria could protect against damage and make these bacteria more tolerant to graphene toxicity than Gram-positive ones. The increased ratio of Gram-negative bacteria in the graphene-intoxicated mice supports this idea. Furthermore, graphene exposure increased the abundance and types of antibiotic resistance genes (ARGs) in the mouse gut, which should be considered an additional health risk of graphene exposure.^125^ On the other hand, graphene and GO have been reported to reduce ARG abundance in anaerobic digestion,^126^ and even GO–silver nanoparticles hybrid composite shows potential for controlling bacteria resistant to antibiotics contributing to nosocomial infections.^127^

As mentioned above, the gut microbiota has the capacity to ferment engineered inorganic carbon nanomaterials, including GO, into organic butyrate. The gut microbiota-governed GO fermentation process was corroborated *in vivo*. Indeed, the level of butyrate in the small intestine and the colon of GO-treated mice for twenty-eight days was 2.9 and 9.7-fold higher, respectively, than in control animals. Moreover, the GO treatment at 200 mg kg^−1^ selectively promoted the growth of butyrate-producing bacteria: Roseburia, Odoribacter, and Ruminococcaceae. However, excessive butyrate derived from microbial GO fermentation inhibited the function (proliferation and differentiation) of intestinal stem cells in mice.^31^ In high-fat diet (HFD)-induced hyperlipidemic mice, oral GO administration at 2.5 mg kg^−1^ day^−1^ for twenty-eight days did not cause any apparent toxic effect on the intestine tissue. Even though the overall abundance of the gut microbiota was not changed, there has been a shift in the intestinal community. The relative abundance of SCFA-producing bacteria was enhanced after seven days of GO exposure. The increase was observed in the *Clostridium* clusters IV/XIVa and *Allobaculum* spp. genera alongside the expression of a key butyrate-producing gene – bacterial butyryl coenzyme A transferase (BcoA). Notably, a microbiome-related decrease in serum total cholesterol and triglycerides and amelioration of the HFD-triggered liver steatosis following GO administration have been suggested. The study gave the idea that increased SCFA production and an enlarged abundance of SCFA-producing bacteria may play a part in the hyperlipidemia-alleviating effect of GO.^29^ A similar effect on *Allobaculum* spp., *Clostridium* cluster IV, and XIVa and the relative abundance of BcoA accompanied by the antihyperlipidemic effects were also observed in mice following 4 week treatment with fullerenol nanoparticles.^128^ Microbiome-regulated bile acid metabolism, SCFA biosynthesis, especially that of propionate and butyrate, and metabolic processes can contribute to host metabolism, including hyperlipidemia management.^129^

There is evidence that graphene nanostructures can protect against colitis.^130,131^ In dextran sulfate sodium (DSD)-induced colitis mice, following oral (4 times, once every three days) administration of 3% solution (1 mg mL^−1^) of graphene quantum dots (GQDs), amelioration of the disease severity was observed. Histological examination revealed loss of crypts, oedema, fibrosis, and immune cell infiltration in colon samples. This was accompanied by decreased blood levels of pro-inflammatory cytokines and splenomegaly compared to colitis mice. Accordingly, GQD treatment protected against body weight loss. Importantly, no signs of toxicity or adverse effect on the gut microbiota were confirmed.^130^ Similar effects *via* modulation of immune cells were observed in colitis mice single injected with GQDs at 15 mg kg^−1^ intraperitoneally.^131^ In both studies, GQD treatment switched the polarisation of macrophages from classically activated M1 to M2 and enhanced intestinal infiltration of regulatory T cells (Tregs). The authors suggested GQDs as an alternative microbiota-friendly anti-inflammatory treatment option against colitis.^130,131^

The results from the *in vitro* and animal studies (Table 1) manifest a diversified impact of graphene-based nanoparticles on bacterial cells and composition, as well as host physiology. The studies present inconclusive outcomes, as the results of the experiments depend on the type of nanoparticles, time of exposure and dosage. Hence, the employment of nanoparticles and the impact of nanoscience raise serious concerns about exposure to living organisms.

**Table 1:** The influence of graphene-based nanomaterials on the gut microbiota by *in vivo* and *in vitro* experiments^a^

Type of nanomaterial, exposure details	Experimental model	Outcomes	References
Pristine graphene at 1, 10, and 100 μg mL^−1^ for 3, 6, and 24 h	Bioreactor, rotary cell culture system, bacterial cultures *of L. acidophilus* (ATCC4356), *B. longum* (ATCC35183), and *E. coli* (ATCC10798)	• ↑ Growth of *L. acidophilus* (24 h)	Lahiani *et al.* 2019 (ref. 20)
Pristine graphene at 1, 10, and 100 μg mL^−1^ for 3, 6, and 24 h	Faecal samples of three healthy male Sprague−Dawley rats aged 4−6 months	• ↑ Aerobic and anaerobic bacteria CFU (3 h)	
		• ↓ Aerobic bacteria CFU (24 h, 100 μg mL^−1^)	
		• ↑ Butyrate-producing genera	
		• Change in the F/B ratio (100 μg mL^−1^)	
		• ↑ Enterobacteriaceae (100 μg mL^−1^)	
GO at 0.05 mg mL^−1^ for 3 days	Pool of bacterial extracts from multiple mice (*n* = 10)/fermentation of GO	• ↑ Butyrate level and ↓ acetate propionate, valeric acid, and caproic acid levels	Cui *et al.* 2023 (ref. 31)
		• ↑ Hexokinase, pyruvate kinase, and pyruvate dehydrogenase activities in the gut bacteria	
		• ↑ Acetyl-CoA, butyryl-CoA, butyrate kinase, and butyryl-CoA:acetyl-CoA transferase levels in the gut bacteria	
GO sheets at 0, 20, 50, and 100 μg mL^−1^ for 2 h	Gut bacterial strains *B. adolescentis*, *L. acidophilus*, *E. coli*, *E. faecalis*, and *S. aureus*	• ↑ Bacterial proliferation	Chen *et al.* 2015 (ref. 102)
		• ↑ *B. adolescentis*, *E. coli*, *S. aureus*, and *L. acidophilus* abundance	
		• ↑ The antagonistic activity of *B. adolescentis* against *E. coli* and *S. aureus*	
GO at 25 and 250 mg L^−1^ GO for 2 h	*In vitro* simulated digestions and colon reactor setup	• ↓ Bacteroidota and ↑ F/B ratio	Couvillion *et al.* 2023 (ref. 103)
		• ↑ Proteobacteria and Firmicutes (25 mg L^−1^)	
		• ↑ Bacteroides, Dysgonomonas, and Parabacteroides (250 mg L^−1^)	
		• ↑ Threonine, isoleucine, methionine, tyrosine, norvaline and tryptophan (25 mg L^−1^)	
		• ↓ Shannon's H index (25 mg L^−1^)	
		• ↑ Shannon's H index (250 mg L^−1^)	
Monolayer graphene, GO, and rGO dietary exposure at 1 μg day^−1^ for 21 days	Wild-type adult zebrafish (*Danio rerio*) aged >6 months	• ↓ Shannon's H index (GR)	Zheng *et al.* 2019 (ref. 108)
		• ↓ Bacteroidetes abundance	
		↑ Lactobacillus abundance, changes in the intestinal tissue and the disintegration of the cell boundaries (GR and GO)	
		• ↓ Pseudomonas abundance (GR, GO, and rGO)	
		• ↓ Aeromonas abundance (GR)	
		• ↑ Cetobacterium abundance (rGO)	
GO dispersion in the environment at 9.14 ± 0.34 mg L^−1^ for 12 days	*Xenopus laevis*, sexually mature	• ↓ Total microbial abundance and shift in the composition	Evariste *et al.* 2023 (ref. 112)
		• ↑ Proteobacteria relative abundance	
		• ↓ Fusobacteria abundance	
		• ↑ F/B ratio	
		• ↑ *B. fragilis* abundance	
GO dietary exposure at 100 mg L^−1^ for 24 h	Wild-type N2 *Caenorhabditis elegans*, pre-treated with *L. bulgaricus* for 12 h	• ↑ Intestinal reactive oxygen species production, ↓ by *L. bulgaricus* pretreatment	Zhao *et al.* 2015 (ref. 117)
		• ↓ The head thrash and body bend, suppressed by *L. bulgaricus* pretreatment	
		• ↑ The mean defecation cycle length, alleviated	
GO, i.g., at 120 mg kg^−1^ every 3 days, for 16 days	C57BL/6 male mice, aged 5 weeks old	• ↓ Body weight	Shen *et al.* 2022 (ref. 121)
		• Damaged intestinal ultrastructure and unevenly, sparsely arranged and shrunken microvilli	
		• ↑ Apoptosis and pyknosis	
		• ↓ β diversity (PCoA)	
		• ↓ Firmicutes, Alistipes, and Prevotellaceae UCG-001 abundance	
		↑ Bacteroidota abundance	
		• ↓ Xanthobacteraceae, Enterobacteriaceae, Enterobacterales and Alistipes	
GO, p.o., 2, 10 or 40 mg kg^−1^ day^−1^, between gestational days 7 and 16	Pregnant ICR mice, aged 7–8 weeks	• ↓ Claudin1 and Occludin mRNA	Liu *et al.* 2021 (ref. 123)
		• ↓ Claudin1 and Occludin	
		• ↓ α diversity indexes	
		• ↓ Cyanobacteria, Chliroflexi and Latescibacteria abundance	
		• ↑ Euryarchaeota abundance	
		• ↑ F/B ratio	
		• ↑ Number of abortions, resorbed embryos and dead fetuses	
		• ↓ Mother and fetus weight	
		• ↑ Damages to placenta histology (10 and 40 mg kg^−1^ day^−1^)	
		• ↓ Phalanx and carpus ossification (40 mg kg^−1^ day^−1^)	
Pristine graphene, i.g., at 1, 10 or 100 μg day^−1^, for 4 weeks	ICR mice, aged 4 weeks	• ↑ Bacterial diversity	Xie *et al.* 2016 (ref. 125)
		• ↓ Lactobacillus and Mycoplasma abundance (1 μg day^−1^)	
		• ↑ Prevotella, Anaeroplasma and Paraprevotella abundance (1 μg day^−1^)	
		• ↑ SOD activity, 8-OHdG level in the liver	
		• ↑ Gram-negative bacteria abundance and types and numbers of ARGs (1 μg day^−1^)	
GO, i.g., at 200 mg kg^−1^ day^−1^, for 28 days	C57BL/6 SPF mice, aged 6–8 weeks/GF mice, aged 6–8 weeks	• ↓ Alistipes and Lactobacillus	Cui *et al.* 2023 (ref. 31)
		↑ Butyrate-producing bacteria: Roseburia, Odoribacter, and Ruminococcaceae	
		• ↑ Butyrate level in the intestine and colon	
		• ↓ Intestinal stem cells, proliferated cells and goblet cells	
GO, i.g., at 2.5 mg kg^−1^ day^−1^, for 28 days	C57BL/6 mice, aged 8 weeks old/HFD- induced hyperlipidaemia	• ↑ Abundance of SCFA-producing bacteria, ↑ abundance of *Clostridium* clusters IV/XIVa and *Allobaculum* spp., and ↑ abundance of butyryl coenzyme A transferase	Li *et al.* 2018 (ref. 29)
		• ↓ Serum and liver total cholesterol and triglyceride levels, ↓ liver steatosis	
GQDs, p.o., 300 μg per mice, 4 times every 3 days	C57BL/6 mice, aged 6 weeks/DSS-induced colitis	• ↓ Disease activity index and histological score	Lee *et al.* 2021 (ref. 130)
		• ↓ Fibrotic regions	
		• ↓ Serum level of IL-6, TNF-α, MCP-1 and IL-12	
		• ↓ Spleen's size	
		• ↓ Proliferation of CD4^+^ T cells	
		• ↑ CD4^+^CD25^+^FoxP3^+^ cells	
		• ↓ Number of *E. coli* BL21 bacterial colonies	
GQDs, i.p., at 15 mg kg^−1^, single dose	C57BL/6 mice, aged 6 weeks/DSS-induced colitis	• ↓ Disease activity index and histological score	Lee *et al.* 2020 (ref. 131)
		• ↑ Colon length	
		• ↓ Serum level of IFN-γ, TNFα, IL-6, and MCP-1	
		• ↑ Serum level of IL-10	
		• ↓ Serum level of IL-2, IL-12, and IFN-γ	
		• ↓ TBX21 and ↑ TGF-β1 in the colon	
		• ↓ TH1/TH17 polarization	
		• ↑ Tregs intestinal infiltration	

a ↓, decrease; ↑, increase; 8-OHdG, 8-hydroxy-2′-deoxyguanosine; ARGs, antibiotic resistance genes; CD4^+^T, CD4 T lymphocytes; CD4^+^CD25^+^FoxP3^+^, type of regulatory T cells; CFU, colony forming unit; DSS, dextran sulfate sodium; F/B ratio, Firmicutes/Bacteroidetes ratio; GF, germ-free; GO, graphene oxide; GQDs, graphene quantum dots; GR, graphene powder; HFD, high-fat diet; IFN-γ, interferon gamma; IL-10, interleukin 10; IL-12, interleukin 12; IL-2, interleukin 2; IL-6, interleukin 6; MCP-1, monocyte chemoattractant protein-1; PCoA, principal coordinate analysis; rGO, reduced graphene oxide; SCFAs, short-chain fatty acids; SOD, superoxide dismutase; SPF, specific pathogen-free; TBX21, T-box transcription factor 21; TGF-β1, transforming growth factor beta 1; TH1/TH17, type 1 T helper cell/type 17 T helper cell; TNF-α, tumor necrosis factor alpha; Tregs, regulatory T cells.

## Possible mechanism of graphene-based nanomaterials' influence on host and gut microbiota

The increased usage of graphene-based nanomaterials in various fields and their presence in the environment might raise concerns about their impact on living organisms. However, the mechanism behind the interaction of graphene-family particles with host cells is not yet fully understood. Based on the results from the previous studies, the impact of graphene depends on dosage, time of exposure, type of material as well as study design.^17,132,133^

**Fig. 3:**
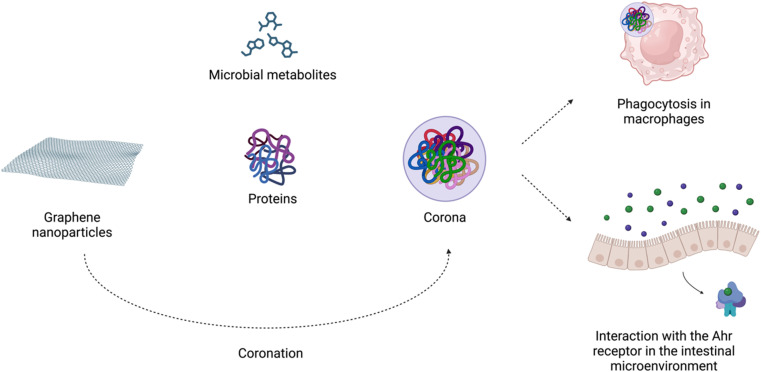
Nano-bio interactions in the intestine environment. Created with https://www.BioRender.com.

Importantly, in biological microenvironments, biomolecules bind to graphene nanoparticles, forming corona. It is widely accepted that the biomolecular complexes determine the biological identity of nanomaterials by presenting key receptor recognition motifs in the corona, which interact with the relevant receptors and drive subsequent biological responses.^134^ Biocorona formation has been demonstrated to affect the uptake, metabolism, immune response, and toxicity of graphene nanostructures.^135^ As Coreas *et al.* 2022 (ref. 135) highlighted, some studies have supported the beneficial effect of the corona protein in reducing the cytotoxicity of these nanomaterials, while others have shown the harmful effects of the corona. Coa *et al.* 2022 (ref. 136) demonstrated that the detrimental effects of GO on *C. elegans* survival were suppressed by the albumin corona due to blocking of its translocation, protecting the intestine and reproductive organs from damage. In adult zebrafish, oral exposure to GO modulated the gut microbiome composition. GO endowed with a ‘corona’ of microbial butyrate triggered type 2 immune responses with the induction of type 2 innate lymphoid-like cells *via* the aryl hydrocarbon receptor (AhR). The authors suggested that graphene-based nanomaterials can modulate the crosstalk between the microbiome and host immune system in an AhR-dependent manner^42^ (Fig. 3).

**Fig. 4:**
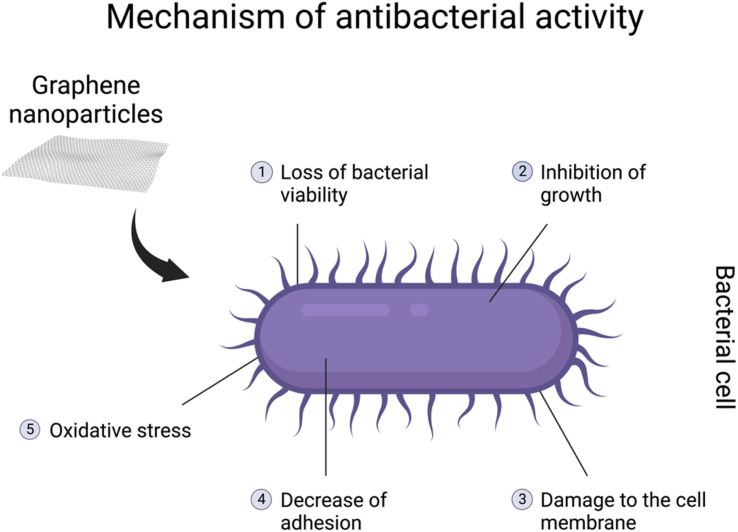
Illustration of the potential antibacterial mechanisms of graphene nanoparticles, which consist of (1) loss of bacterial viability, (2) inhibition of growth, (3) damage to the cell membrane, (4) decrease of adhesion and (5) oxidative stress. Created with BioRender.com.

It has been demonstrated that graphene-based nanomaterials posess antibacterial properties. The antibacterial spectrum might be mostly assigned to GO, rGO, and their nanocomposites.^137–139^ However, most studies are designed to show their properties against pathogenic bacteria genera, including *Escherichia coli, Staphylococcus aureus*, *Streptococcus mutans*, *Porphyromonas gingivalis*, *Candida albicans*, *Pseudomonas aeruginosa*, *Klebsiella* and *Salmonella typhimurium*.^132^ The genera are especially associated with different diseases in various body systems. The antibacterial activity might be related to loss of bacterial viability, damage to the cell membrane, inhibition of growth, or decreasein adhesion^140,141^ (Fig. 4). Due to their characteristics, graphene-based nanomaterials could represent a potential new treatment for pathogenic bacteria. Moreover, due to an overdose and misuse of antibiotics, bacteria might develop specific drug resistance.^132^ Hence, graphene-based particles could be a solution to this growing issue in the healthcare sector. On the other hand, antibacterial properties seem to be a great disadvantage when it comes to symbiotic bacteria and the gut microbiome. As mentioned before, in the *in vivo* studies, graphene-based nanomaterials might interfere with the diversity and functions of the community as well as the abundance of specific bacteria.^121,123,132^ Furthermore, GO can promote the growth of butyrate-producing bacteria capable of incorporating GO into metabolic carbon flow in the gut microbiota to produce beneficial SCFAs. However, excessive butyrate production inhibited the function of neighbouring intestinal stem cells.^31^

Another potential limitation of the practical usage of graphene is the impact and possible toxicity on other cells.^132^ This might also be mainly assigned to GO, rGO and their nanocomposites. Studies indicate that graphene-based nanomaterials might cause oxidative stress.^142^ It is believed that the aggregation of graphene at higher concentrations may occur, elevating the oxidation levels.^143,144^ This could potentially lead to cell cytotoxicity and a shift in the gut microbiota composition.^17^ Research suggests that oxidative stress biomarkers could be elevated after exposure to graphene and its derivatives. This includes levels of oxidative stress, lipid peroxidation, superoxide dismutase, catalase, reactive oxygen species and 8-hydroxydeoxyguanosine.^125,145,146^ Also, the immunotoxicity of graphene exposure could be observed. Studies indicate elevated expression of inflammatory cytokines, TNF-α, IL-1β and IL-6.^147,148^

The antibacterial characteristics and possible nanotoxicity might be a concern in the practical use of graphene-based nanoparticles. Therefore, new methods of synthesis, types of derivatives, modulation of properties, delivery and dosages need to be proposed to improve their functions and impact on bacteria and host cells.^17,132^ However, graphene quantum dots (GQDs) have been studied in terms of their possible protection against oxidative stress. *In vivo* and *in vitro* model studies indicate they are potential antioxidants.^149–151^ In a previous study based on dextran sulfate sodium-induced colitis in mice, GQDs showed a reduction of intestinal damage and colon inflammation. The particles were not harmful to the host cells as well as bacteria.^130^ Hence, GQDs might be the future in graphene nanotechnology and its use in practice. However, more studies need to be performed regarding the dose, administration time and impact on human health.

## Conclusions and prospects

Graphene and its derivatives, thanks to their unique properties, are considered a potential new application in the medical sector. However, only a few studies (Table 1) examined the impact of the exposure and administration of graphene-based nanomaterials on the modulation of host bacterial cells, and gut composition, and their potential health risks. The studies show that graphene-based materials exposure is dosage and time-dependent. Also, different derivatives present various effects on host cells and bacteria. Graphene exposure may cause damage to cell membrane integrity, histopathological alterations, and organ toxicity. Moreover, the route of graphene exposure might influence a shift in the gut microbiota composition. The nanoparticles could alter the functions of the community as well as diversity. Changes could be noticeable in specific phylum or genus abundance. The mechanism of graphene-based nanomaterials' influence on host gut microbiota is not yet fully understood. The antimicrobial properties and induction of oxidative stress are considered. Therefore, there are still concerns about the wide use of these particle nanomaterials.

On the other hand, as we reviewed above, the ability of graphene nanomaterials to affect specific microbes, both pathogenic and beneficial, especially probiotic or SCFA-producing ones, to exert the anti-inflammatory effect alongside good biocompatibility and bioavailability, can be considered in the therapeutical approaches. More recently, the development of graphene-based oral nanomedicine that suppresses intestinal inflammation and modulates the interactions between intestinal microorganisms and the brain for treating IBD has been tackled. TNF-α–siRNA and gallic acid – mediated graphene quantum dots (GAGQDs) encapsulated in bovine serum albumin nanoparticles were demonstrated to effectively treat colitis, maintain bacterial gut microbiota homeostasis, and modulate mood and cognitive dysfunctions in mice. GAGQDs effectively silenced the expression of TNF-α by eliminating intracellular ROS and ensuring the integrity of the TNF-α–siRNA chain.^152^

Since the biological effects of nanomaterials are strongly dependent on their sizes, structures, shapes and surface chemistry and are cell type-dependent,^153^ emphasis should be given to establishing the most desirable synthesis methods, properties, and types of graphene derivatives. Moreover, surface chemistry and coating of graphene-based nanocomposites affecting biomolecule adsorption can be adopted for tuning their cellular impacts. Pre-coating of nanomaterials with host proteins has been reported to enable control of the immune response, either inhibiting clearance by the phagocytes or enhancing complement activation by coating with specific antibodies. Thus, identifying key proteins of interest in the corona is critical for the development of tailored surface modification of graphene nanoplatforms.^154,155^ Accordingly, to optimise exposure to graphene-based nanoplatforms in the context of calibrated delivery and dosing, their time- and dose-dependent activity must be examined in various pharmaco-, toxico-kinetic and dynamic studies. Importantly, more research is required to guarantee safety also with regard to their impact on bacteria and host cells.

On the other hand, as graphene-based nanoparticles may influence the host immune system and microorganisms, reciprocal interactions are also considered. While the enzyme degradation of nanomaterials has been documented in immune cells, including neutrophils and macrophages,^16^ only one study demonstrated fermentation of GO by gut microbes.^31^ Nonetheless, limited available evidence on the environmental degradation of graphene materials and their biotransformation by microbes (reviewed in ref. 42) raises the interest in understanding whether the gut microbiota can digest nanomaterials.

In summary, the effect of graphene-derived nanoparticles on gut microbiota is still under investigation. Due to the variability of the materials tested, cell and biological systems used, and the range of methods for their manufacture and functionalization, it is not possible to draw exact conclusions about the interplay among graphene-based nanomaterials and the microbiome from the collected data available in the current literature. Accordingly, more intense and rigorous studies, especially *in vivo* models, are highly desirable to understand the systemic effect of the exposure of graphene nanoparticles on the gut microbiome and the related health risks alongside potential therapeutic approaches.

## Abbreviations

Ahr: Aryl hydrocarbon receptor
Akt/ERK: v-Akt murine thymoma viral oncogene/extracellular signal-regulated kinase
ARGs: Antibiotic resistance genes
BcoA: Butyryl coenzyme A transferase
CFU: Colony forming unit
CoA: Coenzyme A
DSD: Dextran sulfate sodium
F/B ratio: Firmicutes/Bacteroidetes ratio
GABA: γ-Aminobutyric acid
GAGQD: Gallic acid-mediated graphene quantum dot
GI: Gastrointestinal tract
GO: Graphene oxide
GQDs: Graphene quantum dots
GR: Graphene powder
HFD: High-fat diet
IBD: Inflammatory bowel disease
IL-12: Interleukin 12
IL-1β: Interleukin 1beta
IL-6: Interleukin 6
PCR: Polymerase chain reaction
rGO: Reduced graphene oxide
ROS: Reactive oxygen species
SCFAs: Short-chain fatty acids
TGF-β: Transforming growth factor beta
TNF-α–siRNA: Tumor necrosis factor alpha-small interfering RNA
TNF-α: Tumor necrosis factor alpha
Tregs: Regulatory T cells
Wnt: Wingless-related integration site

## Author contributions

Wojciechowska O. – writing – original draft, review and editing; Costabile A. – writing – review and editing; Kujawska M. – writing – review and editing, supervision, conceptualisation.

## Conflicts of interest

There are no conflicts to declare.
